# Neutropenic Enterocolitis in a Patient With Acute Myeloid Leukemia

**DOI:** 10.7759/cureus.26712

**Published:** 2022-07-10

**Authors:** Erica C Becker, Asiya Tafader, Roopjeet K Bath

**Affiliations:** 1 Internal Medicine, University of Connecticut Health, Farmington, USA; 2 Gastroenterology and Hepatology, University of Connecticut Health, Farmington, USA

**Keywords:** dasantinib, ileocecal syndrome, necrotizing enterocolitis, typhilitis, neutropenic enterocolitis

## Abstract

Neutropenic enterocolitis (NE) is a medical emergency that occurs in neutropenic patients characterized by diffuse circumferential mural thickening predominantly involving the cecum. It is not easily differentiated from various other abdominal conditions (i.e., appendicitis, intussusception, ischemic colitis, small bowel obstruction, pseudomembranous colitis, and viral gastroenteritis), but clinicians should be aware of the diagnostic criteria in order to assist with prompt diagnosis. Although standard treatment has yet to be established, it is necessary to initiate early supportive care to reduce mortality risk. Here we present a case of NE with small bowel obstruction in a patient with acute myeloid leukemia (AML). Clinical findings and CT abdomen/pelvis were consistent with NE. Unfortunately, the patient succumbed to the illness.

## Introduction

Neutropenic enterocolitis (NE) is a severe, life-threatening, inflammatory, and necrotizing condition affecting the ileocecal region and occurs in neutropenic patients [1]. The precise incidence is unknown owing partly to multiple synonyms used to coin this rare condition including typhlitis, necrotizing enterocolitis, and ileocecal syndrome. The underlying pathogenesis is yet to be elucidated; although, the first step is thought to be an intestinal mucosal injury in an immunosuppressed patient [1]. Intestinal edema ensues with the subsequent dissolution of the mucosal integrity allowing for bacterial invasion [1]. Specific criteria have helped providers diagnose typhlitis as other abdominal conditions are not easily differentiated, which can cause a delay in treatment resulting in further complications [2]. Early diagnosis is critical as literature reports mortality rates as high as 50% [3-4]. We present a case of NE with small bowel obstruction in a patient with chronic myeloid leukemia (CML) transformation to acute myeloid leukemia (AML). 

## Case presentation

A 45-year-old Caucasian male with a past medical history of imatinib-resistant CML (diagnosed a decade earlier) on dasantinib for the prior four months presented with generalized weakness, fevers, and abdominal pain associated with non-bloody diarrhea, and severe oral ulcerations rendering him dependent on a liquid diet for several days. The patient was afebrile, blood pressure was 163/75, heart rate was 82, respiratory rate was 16, and he was saturating 99% on room air. A physical exam was significant for diffuse oral ulcers. Laboratory investigations revealed leukocytosis, anemia, and thrombocytopenia (Table 1).

**Table 1:** Laboratory results on admission. MCV, mean corpuscular volume; MCH, mean corpuscular hemoglobin; RBC, red blood cell; MCHC, mean corpuscular hemoglobin concentration

Laboratory test (on admission)	Patient value	Standard value range
White blood cell count (10^3uL)	13.1	43.8-10.6
Neutrophils (%)	59.8	40-70
Immature granulocytes (%)	0	0.0-0.6
Lymphocytes (%)	50	20-50
Monocytes (%)	6	4.0-12.0
Eosinophils (%)	1	0-6
Bands (%)	2	5.0-11.0
Blasts (%)	35	<=0%
RBC count (10^6/uL)	2.18	4.40-5.90
Absolute neutrophil count (10^3/uL)	1	1.4-6.3
Absolute lymphocyte count (10^3/uL)	6.6	0.7-4.5
Absolute monocyte count (10^3/uL)	0.8	0.2-0.8
Absolute eosinophil count (10^3/uL)	0.1	0.0-0.3
Hemoglobin (g/dL)	7.6	13.0-18.0
Hematocrit (%)	23.4	40.0-52.0
MCV (fL)	107.3	80.0-100.0
MCH (pg)	34.9	26.0-34.0
MCHC (g/dL)	32.5	32.0-36.0
RBC distribution width (%)	15.7	11.6-14.8
Platelet count (10^3/uL)	43	150-440
Reticulocytes (%)	0.6	0.8-2.5

His basal metabolic panel was stable with the exception of slightly elevated creatinine of 1.5 mg/dL (standard range of 0.7-1.3 mg/dL) and C-reactive protein of 25.1 mg/dL (standard range of <0.9 mg/dL). Stool study for Clostridium difficile was negative. 

Peripheral smear on presentation revealed 70% blasts consistent with transformation to acute leukemia. He underwent a lumbar puncture after developing severe headache, photophobia, and altered mental status, which revealed blast cells on flow cytometry indicating central nervous system (CNS) infiltration. Bone marrow biopsy showed acute myeloid leukemic blast crisis; thus he was started on intrathecal methotrexate and induction chemotherapy 3-7 regimen with BCR-ABL targeting agent, cytarabine and idarubicin with prophylactic posaconazole and acyclovir. BCR-ABL was not detected by polymerase chain reaction (PCR), therefore, he was transitioned to midostaurin targeting FLT3-TKD. 

**Figure 1:**
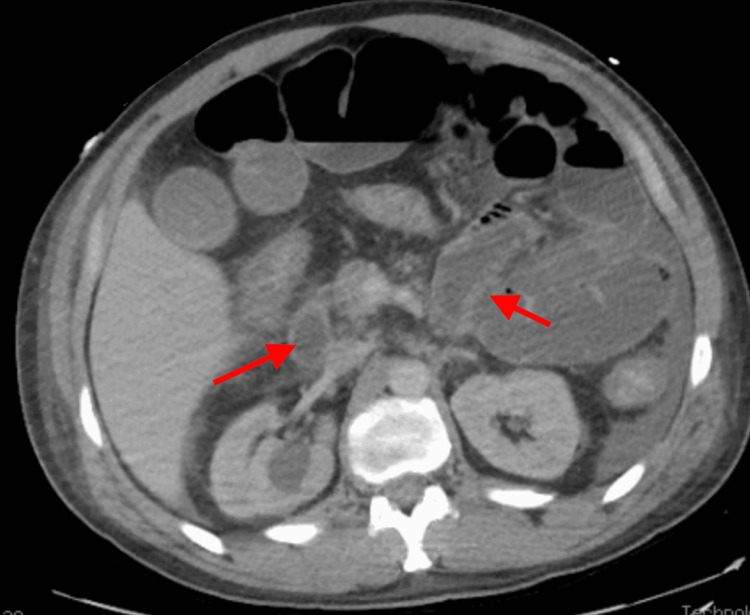
Axial CT abdomen showed diffuse bowel wall thickening with abnormal enhancement (arrows).

**Figure 2:**
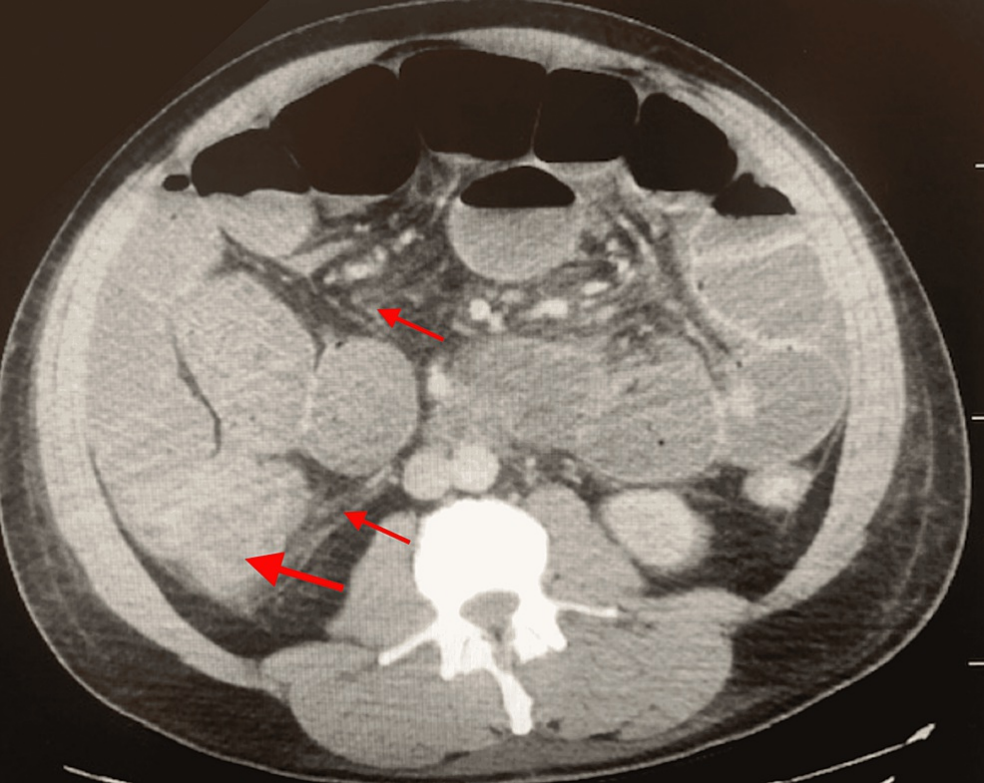
Diffuse circumferential mural thickening predominantly involving the cecum (large arrow) with adjacent pericolonic fat stranding (small arrow).

During his hospitalization, he developed pancytopenia secondary to chemotherapy, neutropenic fevers (while on cefepime, metronidazole, and vancomycin), vomiting, right lower quadrant abdominal pain, and worsening diarrhea. On examination, the abdomen was distended with diffuse tenderness, involuntary guarding, and board-like rigidity. Auscultation revealed hypoactive bowel sounds with tympany on percussion. CT abdomen and pelvis demonstrated acute small bowel obstruction secondary to diffuse circumferential mural thickening predominantly involving the cecum as well as mesenteric, pelvic, and perisplenic ascites concerning for NE (Figures 1-2). 

He became hemodynamically unstable and developed respiratory distress requiring intubation. Thereafter, the patient developed abdominal compartment syndrome leading to decompressive ileostomy without primary surgical closure. Unfortunately, he continued to deteriorate preventing further oncological treatment, and a repeat bone marrow biopsy revealed persistent blasts; thus, he was transitioned to comfort measures only and succumbed to the illness.

## Discussion

In a study involving hospitalized patients with hematological malignancies, solid tumors, and aplastic anemia the incidence was 5.6% [4]. Reports of typhlitis are increasing due to the increasing use of chemotherapy, especially cytotoxic chemotherapy (e.g. cytosine arabinoside and idarubicin) [3]. A high index of suspicion is needed among neutropenic patients undergoing chemotherapy. Abdominal pain in this population has a wide differential.

Elucidation of diagnostic criteria has made the diagnosis of typhlitis less controversial and includes a combination of clinical manifestations and radiological findings [1, 3]. These include the presence of fever, neutropenia (absolute neutrophil count, ANC <500 × 109 cells/L), demonstration of bowel wall thickening by more than 4 mm (transversal scan) over more than 30 mm (longitudinal scan) in any segment by ultrasound or CT, abdominal pain/distention/cramping, diarrhea, and/or lower gastrointestinal (GI) bleeding [1, 3]. Ultrasound is frequently employed in pediatric patients so as to avoid radiation exposure [4]. However, CT provides more detailed information which aids in diagnosis, surgical intervention, and prognosis [4]. 

The predilection for the ileocecal region has been attributed to a combination of cecal distensibility resulting from bowel stasis and limited vascular supply from the terminal branches of the superior mesenteric artery. Distension leads to the further impedance of blood supply and the clinical sequelae of ischemic bowel ensue with disruption of the mucosal integrity. However, there are reports of typhlitis occurring within the terminal ileum, transverse colon, descending colon, and rectum [5]. Pre-existing bowel conditions, such as diverticulitis, tumor infiltrations, and previous surgery also increase the risk of NE [3]. Our case follows the typical predilection with occurrence in the cecum.

Standardized treatment options have not yet been established owing to limited data and clinical trials. Recently, the literature reports a combination of successful methods physicians have utilized for patient care from conservative, nonsurgical management to surgical intervention with right hemicolectomy and ileocolic anastomosis [3]. Surgical intervention was previously considered in all patients, however, current practice is to reserve this for severe cases given the complications associated with surgery in neutropenic and thrombocytopenic patients. Surgical intervention should generally only be considered in the setting of complications such as perforation, abscess formation, persistent GI bleeding, and/or clinical deterioration [6]. Fortunately, improvements in supportive care have led to fewer surgical interventions [3]. Conservative management includes IV fluids, parenteral nutrition, bowel rest, correction of electrolytes, and clotting dysfunction as well as early initiation of broad-spectrum antibiotics [6]. 

Correction of neutrophil counts is recommended and has been associated with increased survival [4, 6]. Granulocyte-colony stimulation factors (G-CSF) have been utilized in severe cases although this remains controversial as there are no randomized control trials available to support its benefit; however, various guidelines have been proposed [4, 7-8]. Some authors have postulated that G-CSF-induced inflammatory responses may theoretically worsen bowel wall integrity and, therefore, its use warrants further study [3]. Glutamine has been used as an immunonutrient to dampen the inflammatory response, although its benefits are unproven [4]. 

## Conclusions

Typhlitis is a medical emergency with a poor prognosis that should be on the differential in neutropenic patients with abdominal pain. Physicians should be aware of the unfavorable complication of chemotherapy agents. Prompt diagnosis is required to prevent unnecessary medical intervention and for a favorable clinical course. The diagnosis significantly relies on reviewing the patient’s symptoms and medical history and correlating the imaging findings with patient symptoms. 
